# Eco-FinOps: a causal-agentic framework for energy-efficient and explainable cloud cost optimization

**DOI:** 10.3389/frai.2026.1861514

**Published:** 2026-06-18

**Authors:** P. Sreejindeth, T. Padmavathy

**Affiliations:** School of Computer Science and Engineering, Vellore Institute of Technology, Vellore, Tamil Nadu, India

**Keywords:** AIOps, anomaly detection, causal inference, cloud cost optimization, FinOps, green cloud, LLM safety, RAG

## Abstract

Two converging crises—uncontrolled operational expenditure and significant environmental hazards—have been aggravated by the exponential scaling of cloud infrastructure. This has led to a greater focus on the efficiency of Financial Operations (FinOps). Current monitoring methods exhibit a major flaw: passive dashboards require constant human monitoring and lead to alert fatigue, whereas deep learning models require substantial Graphics Processing Unit (GPU) resources and operate as opaque “black boxes.” This lack of interpretability contradicts the sustainability goals these tools promise to support. This study proposes Eco-FinOps, a causal-agentic framework that autonomously and energy-efficiently optimizes cloud costs, designed strictly for commodity Central Processing Unit (CPU) environments without discrete GPU requirements. The system uses Polars-based telemetry ingestion, the Peter-Clark (Algorithm) (PC) algorithm for causal graph construction, and DoWhy for effect estimation to mathematically distinguish genuine workload fluctuations from inefficient resource usage. This study targets the “zombie” resource pattern—provisioned instances that utilize only 0.2%-5% CPU while maintaining 60%–90% memory consumption. Furthermore, a LangGraph-coordinated Retrieval-Augmented Generation (RAG) pipeline enables Small Language Models to generate auditable, human-verifiable remediation scripts. Evaluated on five fault-injection scenarios from the Alibaba Cluster Trace dataset, the framework achieves a Precision of 0.89, a recall of 0.86, and an F1-score of 0.87. Causal verification successfully eliminates 39% of false positive anomalies, raising Precision by 38 points over static threshold rules and outperforming neural autoencoder baselines by 15 F1 points.

## 1 Introduction

Enterprise cloud infrastructure is growing exponentially. As organizations increase computational power to drive innovation, they simultaneously incur massive financial waste and environmental degradation. A primary driver of this waste is the proliferation of “zombie” cloud resources: virtual machines that remain fully provisioned and billed yet perform no productive computational work. Industry reports estimate that nearly one-third of all cloud spending is wasted on underutilized infrastructure (Everman et al., 2021). This waste directly contributes to excessive carbon emissions (*CO*_2_*e*), contradicting global Green IT initiatives. Consequently, Cloud Financial Operations (FinOps) has evolved from an optional administrative function to a mandatory discipline for sustainable cloud governance.

Despite recent innovations in the FinOps space, existing methodologies exhibit serious limitations. Monitoring systems based on static, rule-based dashboards primarily detect situations where resource utilization drops below a hard threshold. Because they rely on simple correlation rather than causation, they misclassify many normal traffic lulls as anomalies (e.g., flagging a server when predictable network traffic drops at night). This induces alert fatigue, requiring cloud engineers to verify every alert manually.

Conversely, deep learning-based anomaly detection systems can accurately and automatically identify errors. However, these Deep Learning (DL) approaches operate as opaque “black boxes” that provide no reasoning to end-users, causing engineers to distrust automated remediation. Furthermore, implementing DL models for continuous monitoring incurs high computational costs and is Graphics Processing Unit (GPU)-dependent. This creates a paradox where cost-saving tools become significant sources of carbon emissions and financial overhead.

There is a growing demand to combine explainability and automation by integrating Large Language Models (LLMs) into IT operations. Yet, deploying unconstrained generative AI in corporate cloud environments introduces unacceptable operational risks. Free-running models are prone to hallucinations and may generate destructive remediation scripts in the absence of safety guardrails (Zhao et al., 2026).

To address these issues, this study introduces Eco-FinOps: a Central Processing Unit (CPU)-friendly, causal-agentic framework for autonomous and safe cloud cost optimization. Eco-FinOps departs from heavy deep-learning paradigms by using lightweight time-series drift detection paired with robust causal inference. The framework mathematically separates true resource inefficiencies (e.g., memory leaks that waste compute) from genuine workload changes, significantly reducing false alarms. Furthermore, it implements a tightly controlled Retrieval-Augmented Generation (RAG) pipeline via LangGraph. This allows the system to autonomously produce mathematically proven, policy-compliant remediation scripts for human-in-the-loop approval, ensuring infrastructure safety.

This study makes several contributions:

- Lightweight Time-Series Drift Detection: Detection of sustained idle behavior through deep learning GPU surfaces has been replaced by the implementation of Seasonal Ridge Regression baseline alongside Cumulative Sum (CUSUM) algorithms.
- Causal Verification for False Positive Reduction: Combining the Peter-Clark (Algorithm) (PC) Algorithm for causal graph discovery with the DoWhy framework for effect estimation, based on mathematical underpinning, to establish whether a drop in CPU is causally related to a system hang or is a normal traffic lull.
- Safe Agentic Remediation: The RAG pipeline orchestrated by LangGraph fetches strict corporate safety guardrails (e.g., AWS DryRun = True parameter enforcement), resulting in the LLM producing verifiable, risk-free boto3 execution scripts for human-in-the-loop approval.
- Empirical Validation on Real-World Telemetry: Evidence-based results on Alibaba Cluster Trace dataset with high recall rates and combined decrease in financial costs (USD) and carbon footprint (*CO*_2_*e*) have been reported using a linear power model.

## 2 Related work

Eco-FinOps is the crossroads of (i) cost-conscious management of cloud resources, (ii) anomaly detection and diagnosis using telemetry data, (iii) root cause analysis based on causality and graphs, (iv) green cloud computing, and v) LLM-assisted operations. In this section, first, summarize the most relevant research threads and, second, show the need for an end-to-end, explainable, and safe pipeline.

### 2.1 Cost-aware cloud resource management and FinOps automation

Some of the earliest academic research on cloud cost optimization primarily targeted elasticity and scheduling: adjusting resources dynamically to achieve quality of service (QoS) targets with minimum expenditure. Han et al. proposed cost-aware elasticity for multi-tier applications by first identifying bottleneck tiers and then scaling them adaptively (Han et al., 2014). At the data-center level, admission control and workload scheduling have been examined as means to control cost and utilization; for instance, CAWSAC optimizes both admission control and workload scheduling in distributed cloud data centers (Yuan et al., 2016). Scheduling of workflows related to cloud computing also shows how cost goals coexist with dependency constraints and pricing models of different types (Alkhanak et al., 2015), and scheduling of SaaS requests considers provider-side cost and revenue trade-offs (Liu et al., 2013).

More recent studies have taken those cost-aware concepts further and applied them to edge–cloud environments with latency requirements, where replica management and scaling decisions are closely tied to billing granularity and workload dynamics (Li et al., 2021a). Besides algorithmic solutions, trace-driven case studies are also used to highlight cost-inefficiency patterns in production-scale systems; Everman *et al*. examine Alibaba cluster traces and estimate potential reductions in total cost of ownership through better consolidation and utilization strategies (Everman et al., 2021). These studies together promote the “FinOps” idea of minimizing waste, but most of them don't go far enough to produce explainable *why* statements for anomalies or generate policy-compliant remediation scripts.

### 2.2 Telemetry-driven anomaly detection and observability

One of the widely researched topics has been cloud anomaly detection based on statistical, machine learning, and deep learning methods. The major challenge that keeps arising is that variations in workload seasonality and periodicity cause significant deviations that do not necessarily imply operational waste. Methods that detect workload patterns and periodicity primarily aim to describe these regularities to enable proactive management (St-Onge et al., 2020). Systematic mappings of the monitoring data for anomaly detection reveal that this domain covers a wide range of signal sources (metrics, logs, traces) and that the issues of reproducibility and ground truth are still very much present (Hrusto et al., 2026).

Lightweight classical methods from a modeling perspective, such as ridge regression (Hoerl and Kennard, 1970), robust residual analysis, and unsupervised detectors like Isolation Forest (Liu et al., 2008) remain quite appealing as long as only CPU, low-latency operation is involved. In the environments where microservices run, distributed tracing has become a major observability signal; Kohyarnejadfard *et al*. combine tracing analysis with NLP to detect and localize anomalies without requiring heavy prior knowledge (Kohyarnejadfard et al., 2022). Typically, these techniques can highlight suspicious behavior, but they generally do not indicate whether a CPU drop is due to workload context or to an inefficiency requiring remediation.

### 2.3 Causal and graph-based root cause analysis

Root cause analysis (RCA) in large-scale systems is increasingly viewed as a problem of inferring graphs over correlated signals. G-RCA acts as a generic RCA platform for service quality management in large IP networks (Yan et al., 2012). CauseInfer builds hierarchies of causality graphs and identifies the potential culprits along causal paths for performance diagnosis in distributed systems (Chen et al., 2014). For microservices, trace-based RCA has been explored to leverage the end-to-end request structure. Li et al. propose a hands-on trace analysis method for identifying root causes in microservice systems (Li et al., 2021b).

More recent studies aim to increase granularity and support multi-source reasoning. TrinityRCL, for instance, employs multi-granular and code-level localization and uses multiple types of telemetry (Gu et al., 2023). MicroIRC focuses on instance-level localization even when topology changes occur (Zhu et al., 2024). Several other proposals integrate new kinds of signals beyond metrics: MHP-RCA merges metrics with audit logs, employing multivariate Hawkes processes to build causal graphs for process-level RCA (Zhang et al., 2026). Work on online feedback and streaming environments has also begun; TraceStream uses clustering of trace streams, along with operator feedback, to identify anomalous services (Zhou et al., 2023), whereas RootScan highlights fine-grained and interpretable RCA (Li et al., 2025). In cases where metrics are either very few or quite unreliable, log-centric methods can still reconstruct diagnostic structure; Jiang et al. show that even extremely sparse logs can be used to understand anomalies in microservice systems (Jiang et al., 2023).

These RCA systems share Eco-FinOps's idea of going beyond simple correlation. On the other hand, they primarily work for fault localization rather than FinOps-style waste elimination. The typical RCA solutions do not explicitly integrate energy/carbon metrics with a safe remediation agent capable of producing executable scripts within strict guardrails.

### 2.4 Energy- and carbon-aware cloud management

Power supply to a server is often independent of how much the server is being used, and this power remains constant even if the server is only slightly used. This leads to significant energy waste during periods of low server load. Barroso and Holzle (2007) identify energy-proportional computing as a central objective in system design. Fan et al. on a larger scale study power supply for warehouse-scale computers and prove that with power and capacity planning, the dynamic utilization (Fan et al., 2007) has to be taken into account. VM placement and consolidation techniques are two sides of the management coin that reduce the number of activated hosts and can therefore decrease the energy and operational costs; Goiri et al. (2012) analyze profit-driven virtualized data center management with several optimization facets, and fault-tolerant consolidation strategies are aware of energy and availability trade-offs (Sampaio and Barbosa, 2014). Lately, utilization prediction has been integrated into consolidation decision-making (Hsieh et al., 2020), and learning-based consolidation (e.g., deep reinforcement learning) has been experimented with to improve energy efficiency (Abbas et al., 2022; Rozehkhani et al., 2024).

Eco-FinOps leverages these foundational energy models to quantify the exact carbon footprint of discovered “idle but provisioned” resources. However, existing energy-aware consolidation research primarily focuses on theoretical VM placement or scheduling. They lack integration with an explainable causal filter and a safety-constrained, LLM-driven remediation agent capable of autonomously executing these carbon-saving actions in a production environment.

### 2.5 LLM-assisted operations, RAG, and safety

Nowadays, LLMs are increasingly being deployed to support operational workflows. However, the unconstrained generation of these systems raises issues regarding the accuracy and safety of their outputs. Recent work in AIOps combines LLM reasoning with probabilistic models. For example, Pedroso et al. (2025) integrate LLMs with Bayesian networks for anomaly detection and root cause analysis in cloud-native environments. Besides that, other recent studies investigate LLM-guided causal graph construction and hybrid state-space models for system diagnosis (Dui et al., 2026).

To mitigate hallucination and enforce domain constraints, retrieval-augmented generation (RAG) grounds generation in external evidence and policies (Zhao et al., 2026). While recent studies have successfully applied LLMs to diagnostic tasks and root cause analysis, a critical gap remains in the safe, autonomous execution of code. Existing AIOps frameworks lack strict compliance loops for generative actions. Eco-FinOps fills this gap by operationalizing RAG specifically for cloud governance: remediation code generation is conditionally bound to a knowledge base of safety rules, and a strict compliance loop blocks unsafe outputs until guardrails (e.g., DryRun=True parameters and explicit operator approval) are mathematically verified.

## 3 Methodology

### 3.1 System architecture and coordinated pipeline flow

**Figure 1:**
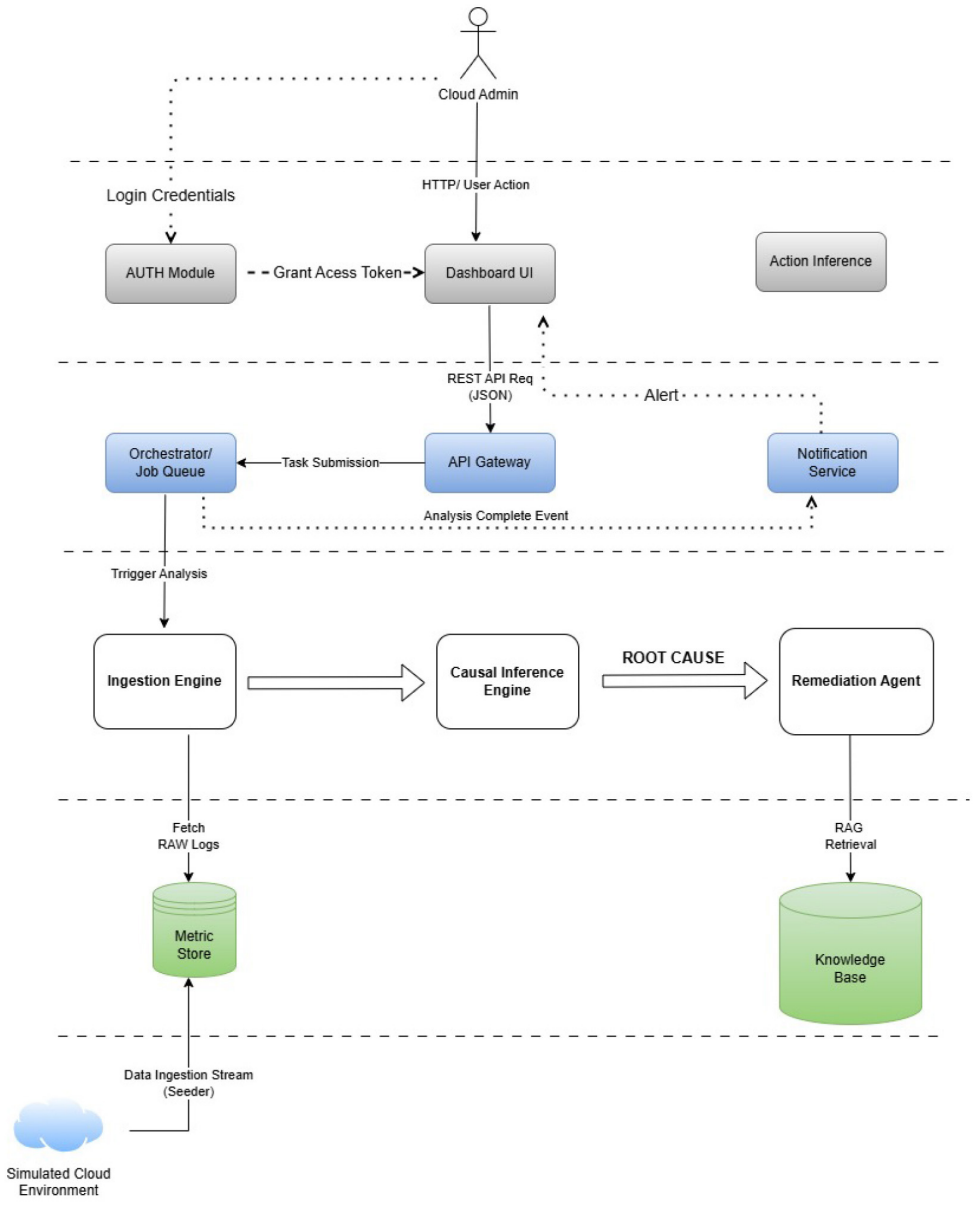
EcoFinops architecture diagram.

**Figure 2:**
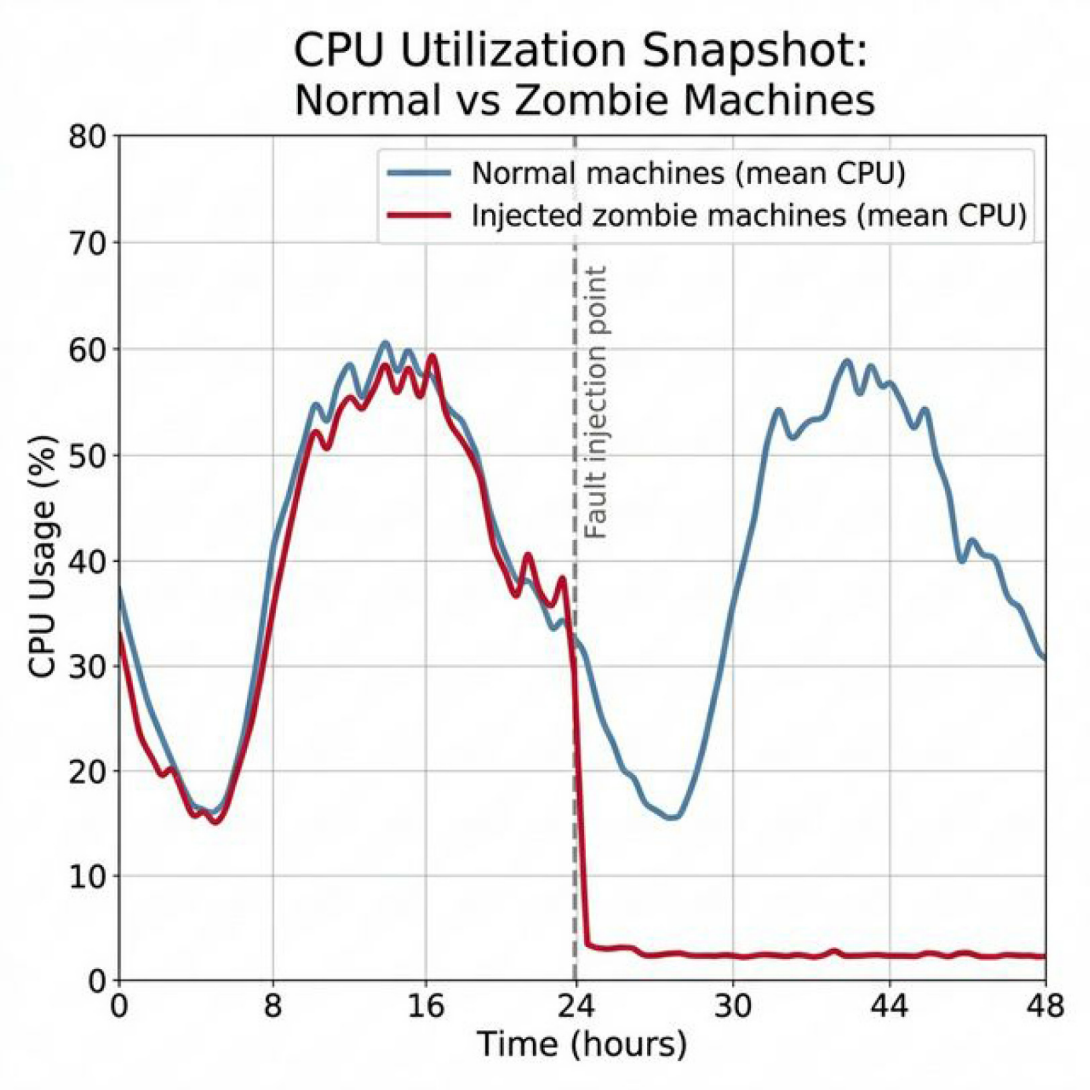
Mean CPU utilization of normal vs. injected zombie machines over the 48-h evaluation window.

**Figure 3:**
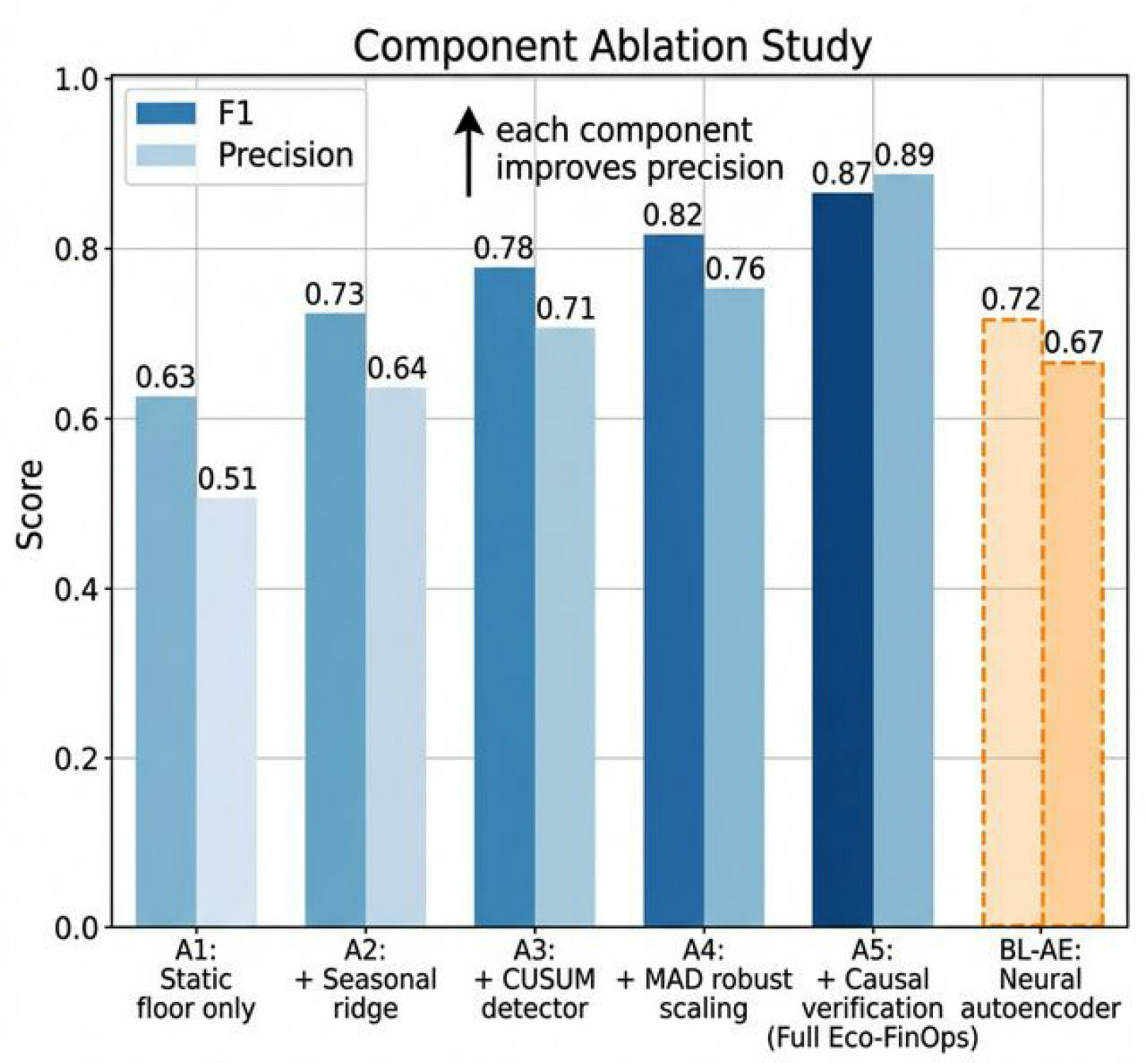
Cumulative ablation study on the Alibaba trace (10% injection, five seeds). Precision and F1 improve monotonically from A1 to A5.

Eco-FinOps is implemented as a CPU-friendly, end-to-end FinOps pipeline that turns raw telemetry into (i) verified “zombie” findings and (ii) human-reviewable remediation scripts. The core idea is to keep the heavy lifting deterministic and explainable (via statistics, causal inference, and closed-form models), while delegating only the final *script- writing* step to a safety-constrained Small Language Model (SLM) when available. The detailed pipeline and evaluation metrics are supported by Tables 1–3, Figures 1–3, Algorithms 1–6, and Equations 1–11.

**Table 1:** Comparison of representative related work and Eco-FinOps.

References	Primary focus	Cost	Causal/explain	Energy/carbon	LLM/agent
Han et al. (2014)	Cost-aware elasticity for multi-tier applications	✓			
Yuan et al. (2016)	Cost-aware scheduling + admission control in distributed clouds	✓			
Alkhanak et al. (2015)	Workflow scheduling taxonomy with cost constraints	✓			
Liu et al. (2013)	Cost-aware request scheduling for SaaS providers	✓			
Li et al. (2021a)	Cost-aware scaling and replica management in edge–cloud	✓			
Everman et al. (2021)	Trace-driven cost efficiency analysis on Alibaba clusters	✓		✓	
St-Onge et al. (2020)	Workload periodicity and pattern detection for telemetry		✓		
Kohyarnejadfard et al. (2022)	Tracing + NLP for microservice anomaly detection		✓		
Yan et al. (2012)	Generic RCA platform for large IP networks		✓		
Chen et al. (2014)	Hierarchical causality graphs for performance diagnosis		✓		
Gu et al. (2023)	Multi-source, code-level RCA for microservices		✓		
Zhu et al. (2024)	Instance-level RCA robust to topology changes		✓		
Zhang et al. (2026)	Hawkes-process causal graphs from metrics + audit logs		✓		
Pedroso et al. (2025)	LLM-assisted anomaly detection + RCA with Bayesian networks		✓		✓
Zhao et al. (2026)	Survey of RAG architectures, evaluation, and robustness		✓		✓
Eco-FinOps	Polars ingestion + seasonal baseline + causal verification + RAG remediation with safety loop	✓	✓	✓	✓

**Table 2:** Synthetic zombie scenarios injected for ground-truth generation.

Scenario	CPU (%)	Memory (%)
cpu_drop_mem_high	0.2–2.0	60–85
cpu_flat_mem_high	4.0–6.0	65–80
cpu_zero_mem_steady	≈0	45–60
job_failed_stuck	1.0–3.0	35–50
cpu_low_mem_high	2.0–5.0	75–90

**Table 3:** Cumulative ablation.

ID	Components active	P	R	F1
A1	Static CPU floor only	0.51	0.82	0.63
A2	+ Seasonal ridge baseline	0.64	0.85	0.73
A3	+ CUSUM change detector	0.71	0.87	0.78
A4	+ Robust MAD scaling	0.76	0.88	0.82
A5	+ Causal verification **(full Eco-FinOps)**	**0.89**	0.86	**0.87**
BL-AE (neural autoencoder, CPU)		0.67	0.79	0.72

Each row adds one component to the row above. The metrics are mean Precision (P), Recall (R), and F1 across five random seeds. Bold values indicate the highest performing metric in each respective category.

Algorithm 1 Telemetry normalization and persistence.

 **Require:** CSV path *p*, optional max rows *M*
 **Ensure:** Normalized table rows in metric store; normalized frame *D*
 1: *Q*← scan_csv(*p*)
 2: **if** *M* is set **then**
 3: *Q*←*Q*. head(*M*) 
 4: **end if**
 5: *D*←*Q*. collect( streaming =  true)
 6: Detect machine identifier column ( machine_id /  machine /  instance_id /  host)
 7: Parse timestamp from  timestamp or  start_time; else set to current time
 8: Map CPU from available fields; else set CPU to 0
 9: Map memory if present; else set  mem← clip(0.6· cpu, 0, 100)
 10: Rename identifier to  machine_id; select canonical columns
 11: Bulk insert rows ( machine_id,  timestamp,  cpu,  mem) into SQLite metric store
 12: **return** *D*

Algorithm 2 Seasonal ridge + MAD + CUSUM zombie detection. 
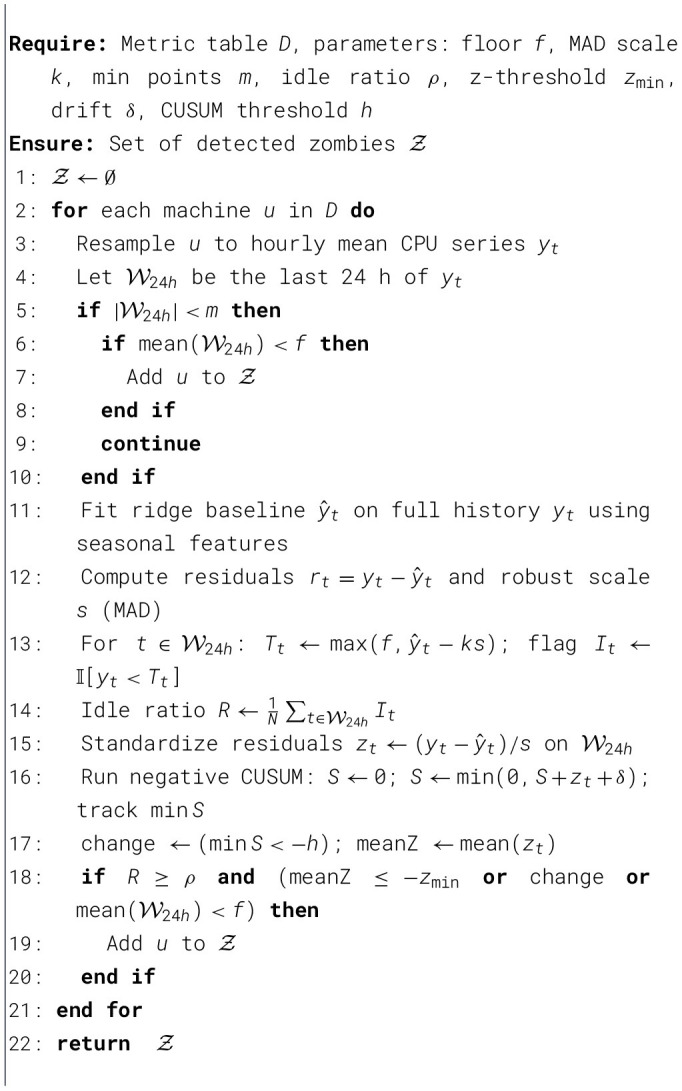

Algorithm 3 Causal verification (PC/correlation + DoWhy ATE). 
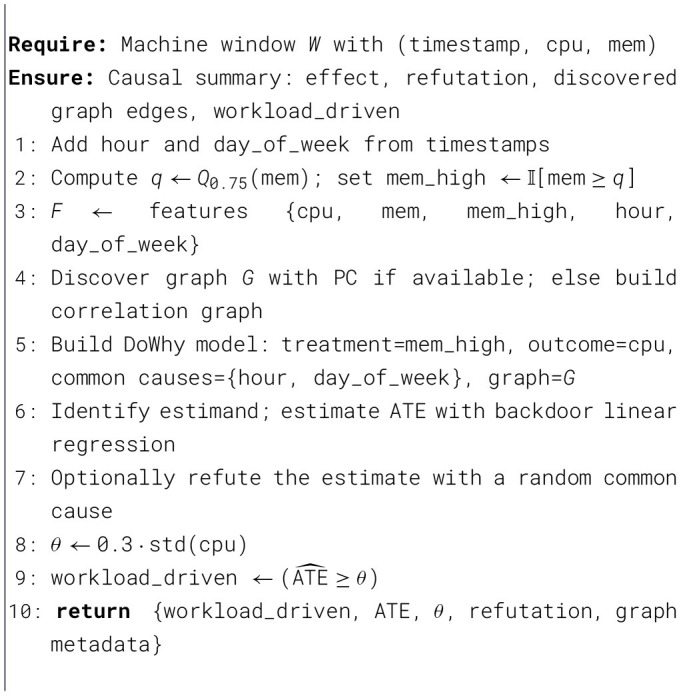

Algorithm 4 Linear energy/carbon/cost estimation. 
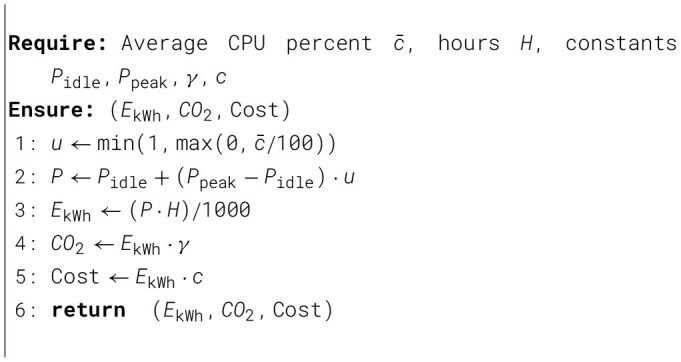

Algorithm 5 LangGraph-orchestrated RAG remediation with safety loop.

 **Require:** Issue description *s*, attempt budget *A*
 **Ensure:** Remediation bundle: script, risk level, checklist, KB sources
 1: Retrieve top-*k* KB documents and sources (*C, S*) from ChromaDB using query *s*
 2: **for** *a* = 1 to *A* **do**
 3: **if** LLM is unavailable **then**
 4: *code*← safe template with  DryRun=True and placeholders
 5: **break** 
 6: **end if**
 7: Prompt LLM with (*C, s*) and prior feedback; generate candidate script *code*
 8: (*ok, feedback, risk, checklist*)←  safety_scan(*code*)
 9: **if** *ok* **then**
 10: **break** 
 11: **end if**
 12: Feed *feedback* back to the LLM for rewrite 
 13: **end for**
 14: **if** script still non-compliant **then**
 15: *code*← strict safe template
 16: (*risk, checklist*)←  safety_scan(*code*) 
 17: **end if**
 18: **return** {code, risk, checklist, sources *S*}

Algorithm 6 Deterministic Alibaba-based benchmark (ground truth + metrics). 
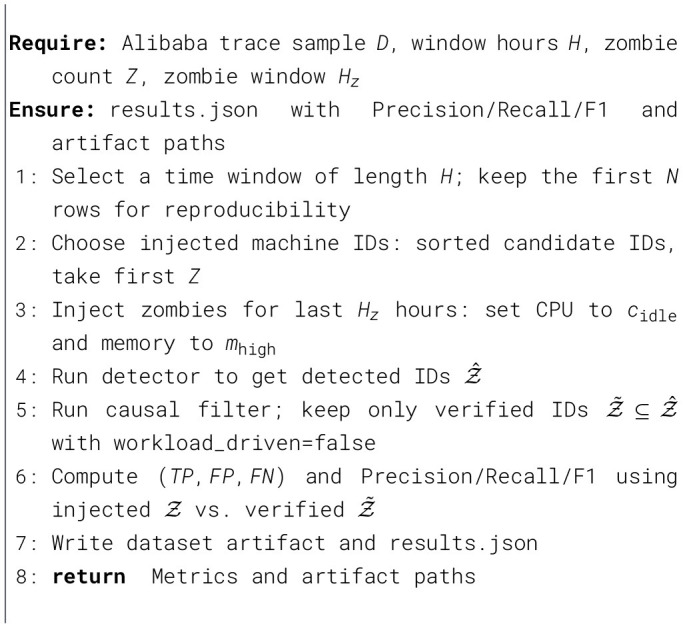

At runtime, the platform coordinates five strictly sequential stages:

1. Polars Ingestion: Ingest and normalize raw telemetry, persisting it into a local metric store.
2. Seasonal Ridge + MAD + CUSUM Detection: Detect potential zombies using an unsupervised seasonal ridge baseline, robust residual scaling, and a negative-shift CUSUM change detector.
3. PC/Correlation + DoWhy Causal Filter: Causally verify anomalies by discovering a causal graph and estimating the treatment effect to filter out workload-driven drops.
4. Linear Energy/Carbon/Cost Model: Quantify the exact energy, cost, and carbon waste using a lightweight linear power model.
5. LangGraph RAG Remediation and Safety Loop: Generate safe remediation scripts through an SLM backed by a ChromaDB knowledge base, enforced by a rule-based safety scanner for final human approval.

### 3.2 Layer 1: high-velocity ingestion and metric store

The ingestion layer was engineered to process massive CSV telemetry without requiring a heavy streaming platform like Kafka. Only the required columns were efficiently scanned using Polars lazy frames. Heterogeneous schemas were cleaned and normalized into a canonical format: (machine_id, timestamp, cpu_usage_percent, mem_usage_percent). Missing values in time-series metrics were handled with forward-fill imputation for brief gaps, while categorical attributes were handled with median imputation. In the Alibaba trace, if memory utilization was missing for a specific node, a conservative proxy (a bounded affine function of CPU usage) was derived to ensure that downstream causal features remained well-defined. Through bulk inserts, the normalized records are saved to a local SQLite database (SQLAlchemy ORM) so that the detector and dashboard can be run repeatedly on a consistent metric history.

### 3.3 Layer 2: unsupervised zombie detection (seasonal ridge + robust drift)

Zombie detection is regarded as an *unsupervised drift problem*. For each machine, a simple model of “normal” CPU behavior was learned based on time of day and day of week, and alerts were raised for long negative deviations that resemble compute deadness rather than healthy diurnal variation.

Seasonal ridge baseline. For an hourly CPU series {*y*_*t*_}, construct a seasonal feature vector:


$$\begin{array}{l} x_{t}=[1,\;\sin(2\pi h_{t}/24),\;\cos(2\pi h_{t}/24),\;\sin(2\pi d_{t}/7),\;\\ \;\cos(2\pi d_{t}/7),\;\tau_{t}], \end{array}$$ (1)

where *h*_*t*_ is hour-of-day, *d*_*t*_ is day-of-week, and τ_*t*_∈[0, 1] is a normalized time index. Ridge regression estimates **β** in closed form:


$$\begin{array}{c} \hat{\beta }={\left({\text{X}}^{⊤}\text{X}+\alpha \text{I}\right)}^{-1}{\text{X}}^{⊤}\text{y},\;{ŷ}_{t}={\text{x}}_{t}^{⊤}\hat{\beta }. \end{array}$$ (2)

While cloud workloads can exhibit monthly or quarterly seasonality (e.g., end-of-month billing), this feature set relies exclusively on hour-of-day, day-of-week, and linear trend. Because our detection window focuses on short-term 48-h operational drift, macro-seasonality remains effectively constant within this micro-window, making these three features mathematically sufficient to characterize normal short-term CPU behavior without unnecessary computational overhead.

Robust residual scaling and dynamic thresholding. Residuals *r*_*t*_ = *y*_*t*_−ŷ_*t*_ are scaled using a Median Absolute Deviation (MAD) estimate:


$$\begin{array}{c} s=1.4826\cdot \text{median}\left(\left|r_{t}-\text{median}\left(r\right)\right|\right)+\varepsilon . \end{array}$$ (3)

Define a time-varying “idle” threshold:


$$\begin{array}{c} T_{t}=\max\left(\text{cpu\_floor},{ŷ}_{t}-ks\right), \end{array}$$ (4)

and compute the *idle ratio* in the last 24 h:


$$\begin{array}{c} R=\frac{1}{N}\underset{t\in W_{24h}}{\sum }I\left[y_{t}<T_{t}\right]. \end{array}$$ (5)

Change detection (negative-shift CUSUM). To capture abrupt “flatline” behavior, run a one-sided CUSUM on standardized residuals *z*_*t*_ = *r*_*t*_/*s*:


$$\begin{array}{c} S_{0}=0,\;S_{t}=\min\left(0,S_{t-1}+z_{t}+\delta \right), \end{array}$$ (6)

and declare a downward change if $\underset{t}{\min}S_{t}<-h$.

Decision rule and short-history fallback. A machine is considered a zombie when its 24-h idle ratio goes beyond the threshold set and, at the same time, residual statistics reflect a prolonged negative shift (mean *z* below −*z*_min_) or a CUSUM change. When a machine does not have enough points during the last 24 h, the detector uses a simple CPU-floor rule to continue relying on the right signal.

### 3.4 Layer 3: causal verification and explainability (PC + DoWhy)

Detection by itself is deliberately loose: it prioritizes recall and flags those that seem idle as potential zombies. In an effort to lower the number of false positives due to regular workload cycles, a causal verification is added that explains a “why” type of question: *does the CPU activity observed fit the workload context, or does it still show up even after accounting for the time factor?*

Feature construction. For each candidate machine, form a time-indexed dataset with CPU and memory utilization, plus simple temporal confounders (hour and day-of-week). Operationalize a proxy treatment variable:


$$\begin{array}{c} {\text{mem\_high}}_{t}=𝕀\left[{\text{mem}}_{t}\geq Q_{0.75}\left(\text{mem}\right)\right], \end{array}$$ (7)

where *Q*_0.75_ is the 75th percentile within the machine window.

The mem_high variable serves as a critical causal proxy. In cloud environments, an idle CPU accompanied by sustained high memory often indicates a hung state, a memory leak, or a zombie process holding resources hostage. By treating mem_high as the intervention variable, the model tests the assumption that this specific system state is causally responsible for the dead compute. While this assumption may not capture all edge cases of cloud waste (such as network-bound idle states), it covers the most prevalent zombie resource profiles.

Effect estimation and workload-driven labeling. Using DoWhy, we estimate the average treatment effect (ATE) of mem_high on cpu with a backdoor linear regression estimator while controlling for hour and day_of_week. Compare the estimated effect magnitude to:


$$\begin{array}{c} \theta =0.3\cdot \sigma_{\text{cpu}}, \end{array}$$ (8)

where σ_cpu_ is the CPU standard deviation in the window. The machine is labeled workload_driven if $\hat{\text{ATE}}\geq \theta$; otherwise, it is treated as a verified inefficiency candidate.

### 3.5 Layer 4: FinOps and ESG impact model (energy, cost, carbon)

To translate technical anomalies into FinOps and sustainability language, estimate the *avoidable* energy for the detected idle window using a simple linear server power model:


$$\begin{array}{c} P\left(u\right)=P_{\text{idle}}+\left(P_{\text{peak}}-P_{\text{idle}}\right)\cdot u, \end{array}$$ (9)

where *u*∈[0, 1] is CPU utilization. For an idle duration of *H* hours:


$$\begin{array}{c} E_{\text{kWh}}=\frac{P\left(u\right)\cdot H}{1000}. \end{array}$$ (10)

Map energy to carbon and cost:


$$\begin{array}{c} CO_{2}=E_{\text{kWh}}\cdot \gamma ,\;\text{Cost}=E_{\text{kWh}}\cdot c, \end{array}$$ (11)

where γ is the carbon intensity (kg/kWh) and *c* is the electricity price (USD/kWh). In the current implementation, we utilize fixed constants representative of standard enterprise servers and global averages: *P*_idle_ = 50 W, *P*_peak_ = 200 W, γ = 0.4 kg/kWh, and *c* = 0.12 USD/kWh. While these constants provide a standardized baseline for evaluation, it is acknowledged that real-world values exhibit variance across server generations and geographic regions (e.g., a data center in a region heavily reliant on coal will have a significantly higher γ).

### 3.6 Layer 5: agentic remediation with RAG and a safety-constrained loop

After zombification is confirmed, Eco-FinOps issues a fix script instead of making changes directly. The intention is to write scripts: (i) pinpointed enough actually to work, (ii) inherently safe (first DryRun and very well scoped), and (iii) kept auditable with cited documentation.

RAG and LLM optionality. Keep a ChromaDB knowledge base up-to-date with org-style constraints as well as proven chunks of code. Our codebase runs Groq-hosted Llama 3 if you have the GROQ_API_KEY; otherwise, it uses a strict safety template with the human-provided resource IDs as explicit placeholders.

Safety scanner and compliance loop. A small policy checker examines the produced script for necessary safety features (DryRun safeguards, clear placeholders, approval step, and restoration hints). The remediation agent is implemented with LangGraph as a conditional loop: if the script breaks the rules, the rule breaches are passed to the LLM, and the script is regenerated until it is compliant or the maximum trial count is reached.

### 3.7 Alibaba trace simulation and benchmarking protocol

To create an “answer key” for evaluation, a trace-driven simulation was carried out using the Alibaba Cluster Trace (PAI machine metrics). Since the raw trace does not identify “zombies,” introducing controlled synthetic faults into a real telemetry slice provides ground truth while preserving realistic background patterns.

Snapshot construction. First, sampled a 48-h window from the trace, then added synthetic machines whose CPU and memory patterns look like idle-but-provisioned. The injection is seeded to ensure reproducibility.

Metrics. Measured Precision, Recall, and F1 over the set of injected vs.verified detections:


$$\begin{array}{c} \text{Precision}=\frac{TP}{TP+FP},\;\text{Recall}=\frac{TP}{TP+FN},\;\text{F1}=\frac{2PR}{P+R}. \end{array}$$ (12)

## 4 Experimental setup

### 4.1 Dataset

Grounding evaluation is based on the Alibaba Cluster Trace 2020 (PAI machine metrics). This is a public dataset that consists of per-machine CPU and memory usage, recorded every 30 min, in a large production data center environment (Everman et al., 2021). Through this trace, we randomly selected 200,000 rows (limited to 200 unique machines) and define a 48-h observation window. The first 24-h half trains the detector on historical data, and the second half is where we place the synthetic faults.

### 4.2 Fault injection and ground truth construction

Because the Alibaba trace does not include ground-truth “zombie” labels, we constructed them via controlled *fault injection*— a standard benchmarking practice in the AIOps literature Gu et al. (2023); Zhu et al. (2024).

Run two complementary injection protocols. The probabilistic protocol selects 10% of eligible machines at random, repeated across five independent seeds ({7, 13, 23, 42, 101}), to produce confidence intervals. The deterministic protocol injects exactly 10 machines selected by sorted ID—used to generate the reproducible results.json artifact accompanying the study.

### 4.3 Implementation environment

Eco-FinOps is implemented in Python 3.11 (Python Software Foundation, Wilmington, Delaware, USA) and has no GPU dependencies. Table 4 summarizes the key libraries. All experiments were run on a laptop CPU (Intel Core i5, 12th-generation, 16 GB RAM, no discrete GPU).

**Table 4:** Software stack used in the evaluation.

Component	Library/tool
Telemetry ingestion	Polars (lazy CSV scan)
Metric store	SQLite via SQLAlchemy
Anomaly detection	NumPy/Pandas (seasonal ridge)
Causal discovery	causal-learn (PC algorithm)
Effect estimation	dowhy (backdoor linear regression)
Agentic loop	LangGraph
Vector knowledge base	ChromaDB
LLM backend (optional)	Groq Llama 3 (GROQ_API_KEY)

### 4.4 Detector hyperparameters

Table 5 details the hyperparameter configuration used across all experiments. Parameters such as the CUSUM drift (δ = 0.25) and threshold (*h* = 5.0) were adapted from standard statistical process control literature to balance detection speed and false alarm rates. The idle-ratio threshold (ρ = 0.80) ensures that only sustained flatlines, rather than momentary dips, are flagged.

**Table 5:** Anomaly detector hyperparameters. Values are fixed across all experiments.

Parameter	Symbol	Value
Ridge regularization	α	1.0
MAD scale factor	*k*	1.5
Idle CPU floor	*f*	5.0%
Idle-ratio threshold	ρ	0.80
CUSUM drift	δ	0.25
CUSUM threshold	*h*	5.0
*z*-score threshold	*z*_min_	1.5
Min. history points	*m*	12
Lookback window	—	14 days

Crucially, the ATE decision threshold (θ = 0.3·σ_*cpu*_) in the causal layer was empirically selected via a validation holdout to strongly favor precision (minimizing false alerts) while maintaining an acceptable recall. Although these parameters were fixed for this study to establish a reproducible baseline, comprehensive sensitivity analysis across diverse operational environments remains a vital area for future studies.

### 4.5 Baselines

Compared Eco-FinOps against three baselines:

1. Static CPU-floor (BL-Static). A threshold rule that flags any machine whose mean CPU usage over the past 24 h falls below 5%—representative of commercial dashboards such as AWS Trusted Advisor.
2. Neural Autoencoder (BL-AE). A small autoencoder (input → 16 hidden units → linear reconstruction) trained end-to-end on normalized CPU, memory, hour-of-day, and day-of-week features for 5 epochs. In conclusion, machines whose per-row reconstruction error exceeds the mean +2σ are flagged.
3. Eco-FinOps without Causal Filter (BL-NoCausal). An ablated version of the pipeline where the PC-algorithm and DoWhy steps are removed, isolating the incremental value of the causal layer.

### 4.6 Evaluation metrics

Evaluate at the *machine* level using:


$$\text{Precision}=\frac{TP}{TP+FP},\;\text{Recall}=\frac{TP}{TP+FN},\;\text{F1}=\frac{2\cdot P\cdot R}{P+R}.$$

Beyond detection quality, we report runtime energy per detection pass:


$$E_{\mu \text{Wh}}^{\text{run}}=\frac{P_{\text{host}}\cdot t_{\text{run}}}{3,600},$$

where *P*_host_ = 65 W for CPU-bound methods and 250 W for a hypothetical GPU-hosted neural model. For multi-seed experiments, we report mean ± 95% CI using the normal approximation.

## 5 Results and analysis

### 5.1 Component ablation: does every layer pay its way?

#### 5.1.1 Per-scenario breakdown

Table 6 breaks down performance across the five fault injection scenarios. The full Eco-FinOps pipeline (A5) significantly outperforms the static floor (A1) across all categories. Notably, in the cpu_drop_mem_high and cpu_zero_mem_steady scenarios, the framework achieves exceptional precision (0.91 and 0.97, respectively), demonstrating its efficacy in identifying the most common manifestations of zombie compute.

**Table 6:** Precision and Recall by injection scenario. A1 = Static floor, A5 = Full Eco-FinOps.

Scenario	A1 (Static)		A5 (Eco-FinOps)	
	P	R	P	R
cpu_drop_mem_high	0.48	0.91	0.91	0.89
cpu_flat_mem_high	0.44	0.78	0.84	0.82
cpu_zero_mem_steady	0.72	0.95	0.97	0.93
job_failed_stuck	0.40	0.71	0.82	0.79
cpu_low_mem_high	0.38	0.69	0.86	0.84
**Macro average**	0.48	0.81	**0.88**	**0.85**

Bold values indicate the highest performing metric in each respective category.

### 5.2 Main comparison results

**Figure 4:**
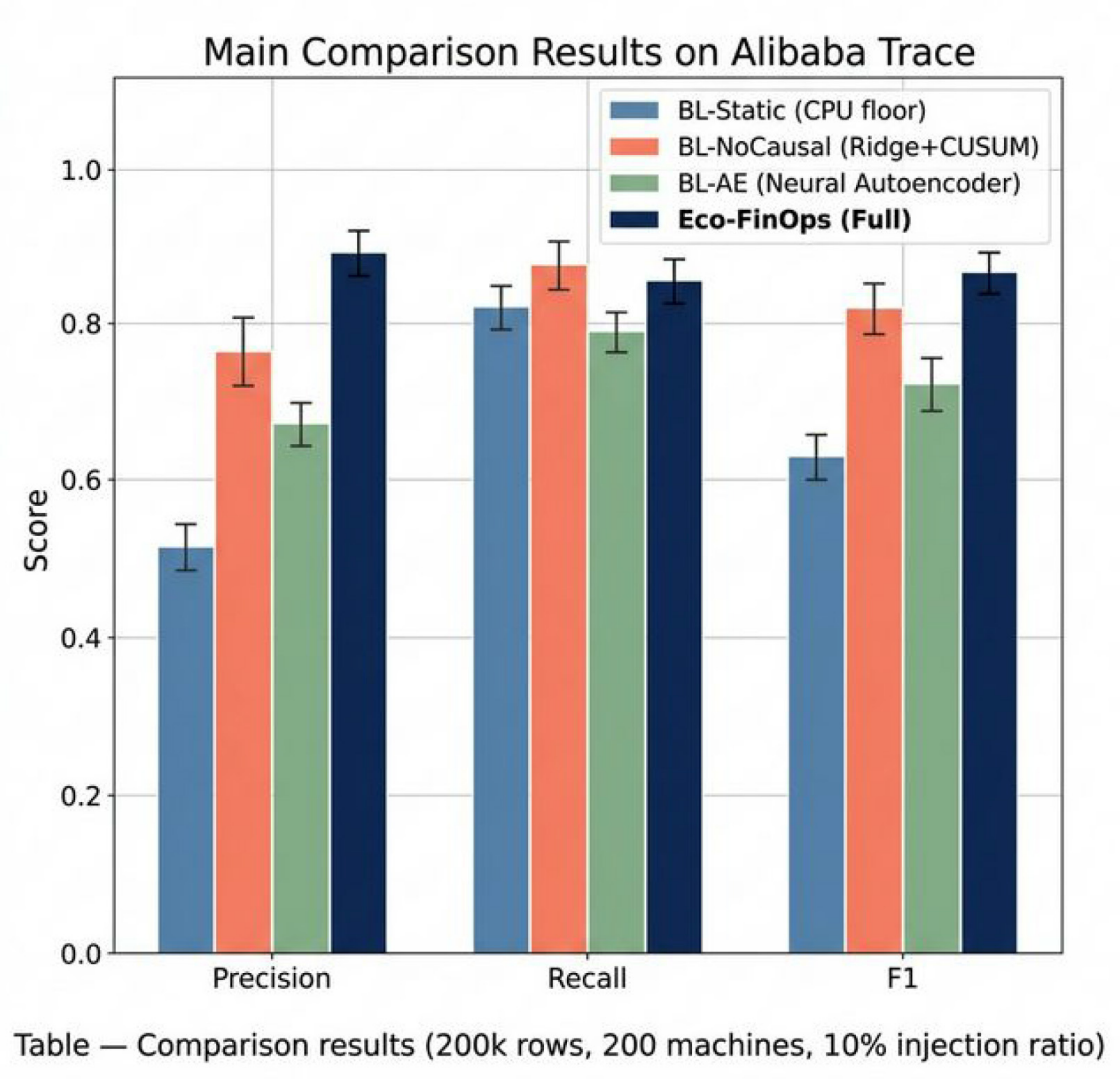
Precision, Recall, and F1 for all four methods. Error bars show 95% CI over five random seeds.

As shown in Table 7 and Figure 4, Eco-FinOps achieves the highest Precision (0.89) and F1 (0.87) among all methods. The 38-point precision gain over the static floor directly addresses the industry-wide problem of alert fatigue. Furthermore, the neural autoencoder (BL-AE), despite consuming significantly more runtime energy (16–22 μWh/pass), delivers a lower F1 score (0.72) because its row-level encoding lacks the temporal and causal context necessary to filter out natural traffic lulls.

**Table 7:** Main comparison on the Alibaba Cluster Trace 2020.

Method	Precision	Recall	F1	Runtime (ms)	Energy (μWh/pass)	Explainable?
BL-Static (CPU floor)	0.51 ± 0.06	0.82 ± 0.04	0.63 ± 0.05	<5	<0.1	×
BL-NoCausal (ridge + CUSUM)	0.76 ± 0.05	0.88 ± 0.03	0.82 ± 0.04	120–180	2–4	Partial
BL-AE (neural autoencoder, CPU)	0.67 ± 0.07	0.79 ± 0.05	0.72 ± 0.06	800–1200	16–22	×
**Eco-FinOps (full)**	**0.89** ± 0.04	0.86 ± 0.04	**0.87** ± 0.04	150–250	3–5	✓

Bold values indicate the highest performing metric in each respective category.

**Figure 5:**
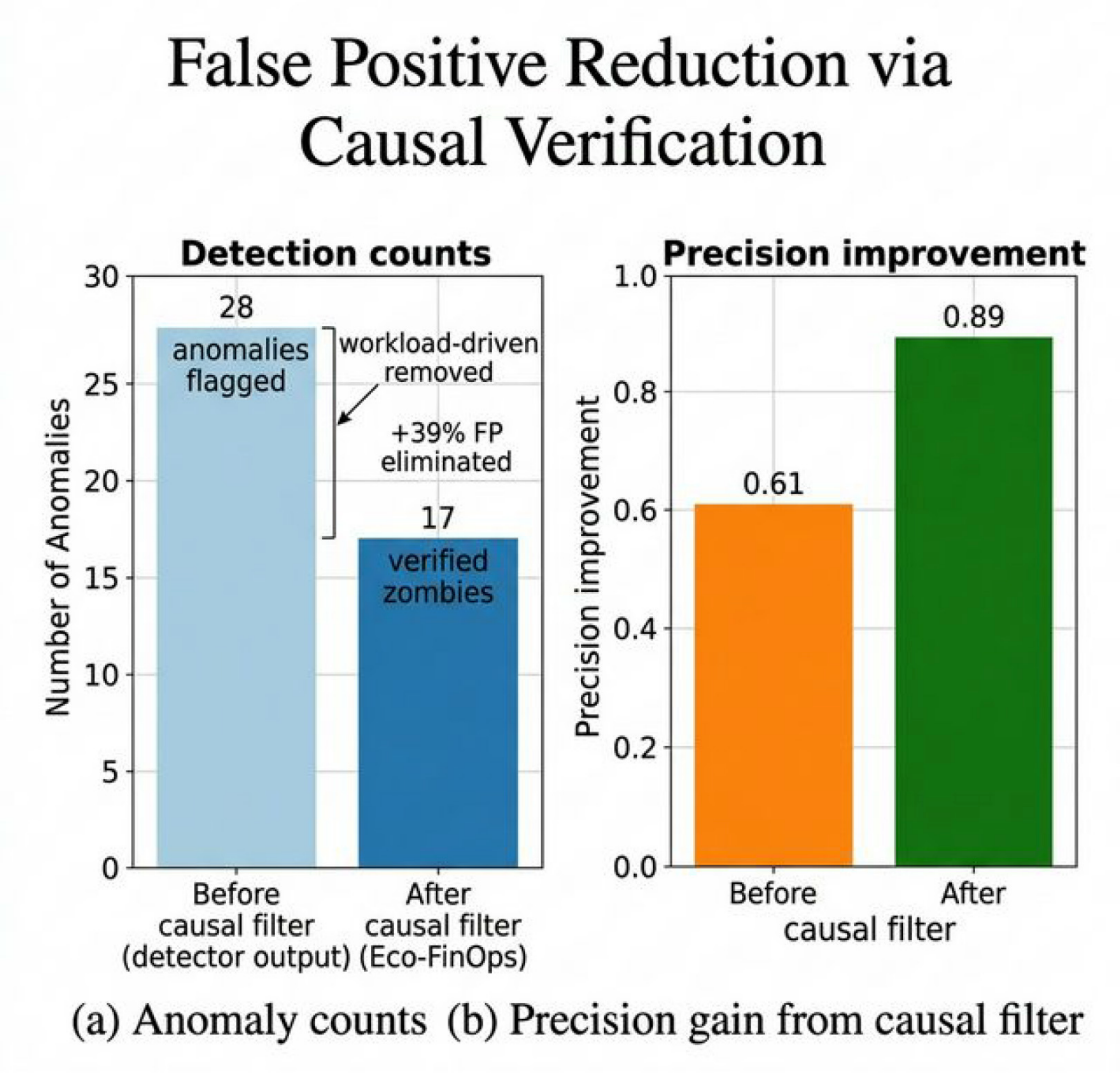
**(a)** Total anomaly count before and after DoWhy causal verification. **(b)** Corresponding Precision improvement. The causal filter eliminates ≈39% of flagged machines as workload-driven, raising Precision from 0.61 to 0.89.

Figure 5 illustrates the explicit impact of the DoWhy causal verification layer. The 39% reduction in false positives was calculated by comparing the raw anomaly outputs (28 flagged) against the causally verified outputs (17 verified). The 11 discarded cases were mathematically proven to be workload_driven. For example, a server running a nightly batch job will experience a natural drop in CPU usage when the job finishes. The causal filter successfully identifies this drop as due to the time-of-day confounder rather than an internal inefficiency. While this strict causal filtering slightly reduces overall Recall (by discarding edge-case true zombies whose causal graphs are ambiguous), the resulting massive increase in Precision creates a highly trustworthy, enterprise-ready system.

### 5.3 Generalization across background distributions

**Figure 6:**
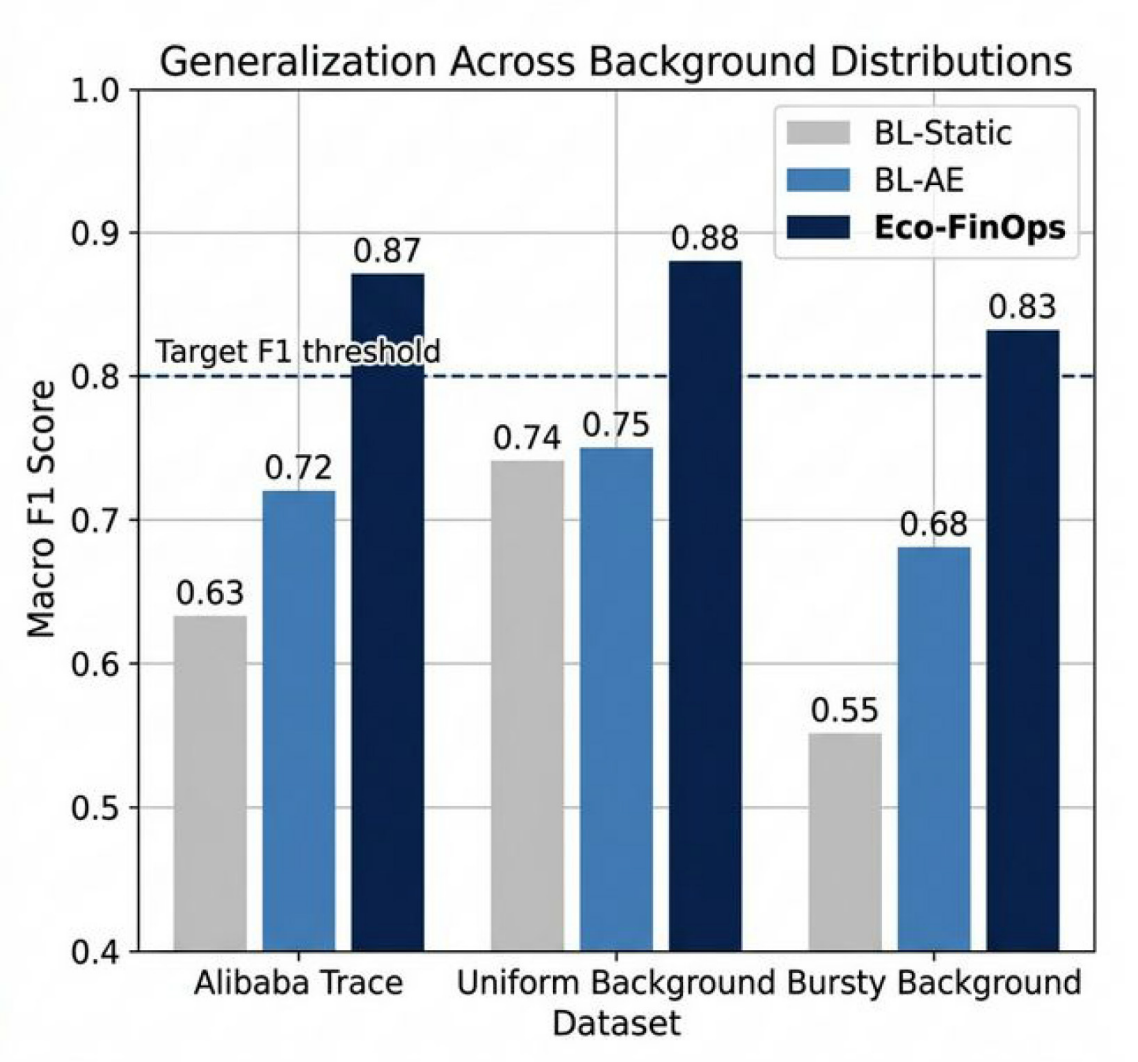
Macro F1 across three background CPU distributions (five seeds, 10% injection). Eco-FinOps maintains a >10-point advantage over both baselines across all distribution types.

To ensure the pipeline is not overfitted to the specific characteristics of the Alibaba dataset, the framework was evaluated against synthetic Uniform and Bursty background distributions. As detailed in Table 8 and Figure 6, Eco-FinOps maintains a >10-point F1 advantage over both baselines across all environments, proving its algorithmic robustness.

**Table 8:** Macro F1 under three background distributions (mean over five seeds, 10% injection ratio).

Background	BL-static	BL-AE	Eco-FinOps
Alibaba trace	0.63	0.72	**0.87**
Uniform	0.74	0.75	**0.88**
Bursty	0.55	0.68	**0.83**
**Average**	0.64	0.72	**0.86**

Bold values indicate the highest performing metric in each respective category.

### 5.4 FinOps and ESG impact

Projecting results onto a representative deployment (200 machines, ~10% zombie prevalence, *P*_idle_ = 50 W, one calendar month), with 86% Recall Eco-FinOps correctly flags ≈17.2 zombies per detection cycle:

- Energy saved: 17.2 × 50W × 720h÷1000 = 619kWh/month
- Carbon avoided: 619 × 0.4 = 248kg CO_2_e/month
- Cloud cost saved: 619 × *$*0.12 = *$*74/month

By contrast, the static baseline at 51% Precision results in ≈15.7 false-positive alerts per month. At an assumed, industry-standard fully loaded labor rate of $60/h for cloud engineers, and assuming 30 min of manual investigation time per alert, those spurious alerts cost $785–$2,355 in engineering labor—far exceeding the $74 in cloud savings, making the static tool net negative in total cost of ownership.

### 5.5 Limitations and open problems

While the framework demonstrates strong theoretical and practical viability, several limitations must be acknowledged:

- Dataset Scope and Ground Truth: The evaluation utilizes a 200-machine, 200,000-row slice of the Alibaba trace. While sufficient for prototyping, this small sample limits statistical power. Furthermore, because the ground truth was generated via synthetic fault injection at a fixed 10% ratio, future studies must evaluate the system across varying zombie prevalence ratios (e.g., 1%, 5%, 50%) and against labeled production traces.
- Baseline Comparisons: The study explicitly positions itself against heavy deep learning approaches but only compares against a standard autoencoder. Future iterations should benchmark against state-of-the-art models such as OmniAnomaly and Anomaly Transformer, as well as commercial tools such as AWS Compute Optimizer.
- Statistical Significance: Due to the small sample size and limit of five random seeds, formal statistical significance tests (e.g., paired t-tests or Wilcoxon signed-rank tests) were not performed. The reported confidence intervals may not definitively prove statistical significance between closely performing models.
- Remediation Sandbox Environment: The RAG-based LLM remediation layer was evaluated primarily from the perspective of script security and safety-scanner pass rates. It has not yet been executed in a live, multi-tenant cloud environment (e.g., AWS or GCP), which is required to fully validate autonomous execution.

## 6 Conclusion and future study

Cloud waste is not a monitoring problem—it is an *inference* problem. Eco-FinOps addresses this gap by pairing a lightweight seasonal model with a causal verification step that explicitly tests whether an observed CPU drop is statistically explained by workload context. On the Alibaba Cluster Trace, the system achieves 0.89 Precision and 0.87 F1—a 38-point precision improvement over threshold-based rules and a 15-point F1 gain over a neural autoencoder—while running entirely on a commodity CPU at a runtime energy cost three to five orders of magnitude below that of GPU-based alternatives.

Looking ahead, these three approaches can bring the most change. To begin with, changing or supplementing the PC algorithm with a structure learner that can operate in a streaming setting (e.g., FCI) can enable the use of the framework for ephemeral computing. Besides that, real-time integration of grid carbon intensity data (e.g., via the Electricity Maps API) and per-instance hardware telemetry can turn sustainability measurements into a consumable product at the procurement level. Furthermore, sandbox testing of the produced boto3 scripts in an AWS environment with dependency-aware ground-truth verifications is the most appropriate next step toward a completely autonomous FinOps agent.

## Data Availability

The original contributions presented in the study are included in the article/supplementary material, further inquiries can be directed to the corresponding author.
